# Long-Term Follow-Up of Patients With Idiopathic Pulmonary Fibrosis Treated With Pirfenidone or Nintedanib: A Real-Life Comparison Study

**DOI:** 10.3389/fmolb.2020.581828

**Published:** 2020-09-04

**Authors:** Paolo Cameli, Rosa Metella Refini, Laura Bergantini, Miriana d’Alessandro, Valerio Alonzi, Carlo Magnoni, Paola Rottoli, Piersante Sestini, Elena Bargagli

**Affiliations:** Respiratory Diseases Unit, Department of Medicine, Surgery and Neurosciences, University of Siena, Siena, Italy

**Keywords:** idiopathic pulmonary fibrosis, real-life, pirfenidone, nintedanib, disease progression, mortality

## Abstract

**Background:**

Pirfenidone and nintedanib are the sole pharmacological therapies currently approved for idiopathic pulmonary fibrosis (IPF). Limited comparison data is available in literature, despite they are both prescribed for mild-to-moderate disease. Here, we describe our almost 10 years real-life experience with antifibrotic treatment to investigate potential differences in terms of efficacy.

**Population and Methods:**

We retrospectively recruited patients diagnosed with IPF and treated with pirfenidone or nintedanib at Siena Referral Center. Clinical, functional, safety and radiological data was collected at baseline and during the follow-up, according to our Center protocol.

**Results:**

We retrospectively recruited 263 IPF patients (139 treated with pirfenidone and 124 with nintedanib) in the study. After 885.3 ± 559.5 days of observation, the median survival was 1224 days. No significant differences were found between pirfenidone and nintedanib in terms of survival and time to decline of forced vital capacity >10% (*p* = 0.8786 and *p* = 0.1677, respectively). A smaller lung diffusion for carbon monoxide (DL_*CO*_) decrease was found after 1 year of therapy with nintedanib in respect to pirfenidone (*p* = 0.0167). Overall, 21 patients permanently discontinued antifibrotic treatment due to side effects (14 with pirfenidone, 7 with nintedanib); no fatal adverse events were recorded.

**Discussion:**

Our results showed a similar effectiveness between pirfenidone and nintedanib in terms of mortality and functional disease progression. Both drugs confirmed their good tolerability profile and no new safety alerts were observed. Nintedanib was associated with a smaller reduction of DL_*CO*_ after 1 year of follow-up compared with pirfenidone, maybe due to its antiangiogenic properties.

## Introduction

Idiopathic pulmonary fibrosis (IPF) is a chronic fibrosing lung disease, characterized by dry cough and progressive exertional dyspnea, invariably leading to respiratory failure and death within 3–5 years of diagnosis (Raghu et al., 2018). Pathogenesis is characterized by aberrant overexpression of profibrotic pathways, triggered by oxidative-nitrosative stress and alveolar epithelial injury refractory to anti-inflammatory and immunosuppressant treatments (Wolters et al., 2014; Sgalla et al., 2018; Cameli et al., 2019, 2020). Today, an “antifibrotic” approach is widely accepted and the only drugs approved for the treatment of IPF are pirfenidone and nintedanib. Randomized clinical trials of both drugs have demonstrated similar efficacy in slowing functional deterioration (expressed as decline in FVC percentages) and the risk of hospitalization for respiratory problems (Noble et al., 2011; King et al., 2014; Richeldi et al., 2014). Real-life studies have confirmed the effectiveness of pirfenidone and nintedanib in slowing the progression of IPF, suggesting an improvement in survival rate (Fisher et al., 2017; Margaritopoulos et al., 2018; Lancaster et al., 2019) but without any substantial effect on quality of life.

Few evidences regarding the comparison of the efficacy of pirfenidone and nintedanib are currently available in the literature. A metanalysis published in 2016 failed to find any significant differences in safety or concerning progression between the two drugs, whereas nintedanib appeared to be more effective in reducing acute exacerbations and mortality risk (Rogliani et al., 2016). Two other retrospective observational studies confirmed the substantial similarity of the two antifibrotic drugs in terms of effectiveness and tolerability, albeit with a relatively short follow-up (Bargagli et al., 2019; Cerri et al., 2019).

Here we describe our almost 10 year experience with antifibrotic treatment at our Regional Referral Center for Rare Lung Diseases and lung transplant, assessing the effectiveness and safety of pirfenidone and nintedanib in a real-life cohort of IPF patients monitored over a long observation period.

## Materials and Methods

### Study Population and Design

All IPF patients treated with pirfenidone and nintedanib at the Regional Referral Center for Rare Lung Diseases, Siena, from 2011 to June 2019 were retrospectively enrolled in the study. Diagnosis of IPF was according to international guidelines (Raghu et al., 2011, 2018). High-resolution computed tomography (HRCT) of the chest was performed in all patients for diagnostic purposes. The diagnosis of IPF was confirmed by multidisciplinary discussion. Demographic and clinical data, including comorbidities, family history, lung function parameters and radiological features were collected from medical records and entered in an electronic database for statistical analysis. We included serial assessment of all clinical and functional data collected regularly every 12 months. If available, we also included lung function test (LFT) parameters 1 year before starting antifibrotic therapy. Survival data and all causes of death were obtained by systematic revision of the medical records of our Center. Adverse effects related to treatment were also collected.

Patients were considered lost to follow-up in the case of death, lung transplant or interruption of treatment. Functional deterioration was defined as a decrease in FVC of 10% or more.

The study was conducted according the principles of the Declaration of Helsinki. All patients gave their written informed consent to participation in the study. The study was approved by our local ethical committee C.E. A. V. S. E. (code number 180712).

### LFTs

Lung function tests were performed according to ATS/ERS standards (Miller et al., 2005; Graham et al., 2017) using a Jaeger body plethysmograph with corrections for temperature and barometric pressure. Forced expiratory volume in the first second (FEV1) and lung diffusing capacity for carbon monoxide (DLco) were recorded.

### Statistical Analysis

Data was expressed as mean ± standard deviation, unless otherwise specified. Parametric tests (*T*-test and one-way ANOVA) were used to compare groups. Statistical analysis and graphs were performed and plotted using GraphPad Prism version 5.0 software for Windows (GraphPad Software, La Jolla, CA). Unadjusted survival and disease progression outcome estimates were obtained using Kaplan-Meier curves. Time-to-event endpoints were compared using a two-sided log-rank test. A *p* ≤ 0.05 was considered significant.

## Results

### Clinical and Functional Features of Study Population

A total of 263 patients (203 males, age 70.9 ± 8.6 years) affected with IPF were included in the study. Demographic characteristics, clinical-functional data and radiological features of the population are reported in Table 1. As expected, the majority of our patients were males and current/former smokers. On average, functional assessment at baseline showed mild restrictive impairment of lung volumes, associated with a moderate reduction in DLco percentages. DLco at baseline was available for 203 patients (105 treated with pirfenidone), who were able to perform the procedure and were not on oxygen therapy.

**TABLE 1:** Demographic, functional and radiological features of the study population.

	Study population	Pirfenidone	Nintedanib	*p*-value*
***N*°**	263	139	124	
**Male (%)**	203 (77.1)	107 (76.9)	96 (77.4)	0.8360
				Chi square: 0.04285, df 1
**Age (years)**	70.9 ± 8.6	68.1 ± 7.7	74.1 ± 8.5	<0.0001
**Smoking history (pack/year)**	24.1 ± 17.9	24 ± 19.5	24.7 ± 12.8	0.8953
Current smoker (%)	9 (3.4)	5 (3.6)	4 (3.2)	0.1265
				Chi-square: 4.134, df 2
Former smoker (%)	168 (63.8)	81 (58.2)	87 (70.1)	0.1265
				Chi-square: 4.134, df 2
Never smoker (%)	86 (32.6)	53 (38.1)	33 (26.6)	0.1265
				Chi-square: 4.134, df 2
**Familial history positive for ILD (%)**	53 (20.1)	29 (20.8)	24 (19.3)	0.8778
**Time from diagnosis to treatment (months)**	7.8 ± 14.9	8.6 ± 12.4	5.1 ± 6.6	0.1512
**Previous treatments**				
Steroids (%)	59 (22.4)	35 (25.1)	24 (19.3)	0.3008
N-acetyl cysteine (%)	20 (7.6)	15 (10.7)	5 (4)	0.0601
Azathioprine (%)	3 (1.1)	3 (2.1)		n.a.
**Comorbidities**				
Emphysema (%)	35 (13.3)	20 (14.3)	15 (12.1)	0.7165
Lung cancer (%)	7 (2.6)	3 (2.1)	4 (3.2)	0.9055
Pulmonary arterial hypertension (%)	18 (6.8)	10 (7.1)	8 (6.4)	1.000
Gastroesophageal reflux disease (%)	119 (44.8)	67 (48.2)	52 (41.9)	0.3232
Arterial Hypertension (%)	167 (63.4)	87 (62.5)	80 (64.5)	0.7095
Ischemic heart disease (%)	79 (30)	41 (29.4)	38 (30.6)	0.8941
Diabetes mellitus (%)	32 (12.1)	20 (14.3)	12 (9.6)	0.1899
**PFTs**				
FVC% predicted value	78.2 ± 20.1	82.6 ± 19.7	73.1 ± 19.9	0.0004
(l)	(2.5 ± 0.7)	(2.7 ± 0.8)	(2.3 ± 0.7)	0.0002
FEV1% predicted value	81 ± 19	85.5 ± 18.6 (2.2 ± 0.6)	76.2 ± 18.9 (1.8 ± 0.5)	0.0004
(l)	(2 ± 0.6)			0.0001
FEV1/FVC	80.9 ± 7.6	81 ± 7.5	80.8 ± 7.7	0.8834
TLC% predicted value	76.4 ± 17.1	80.5 ± 17.2 (4.7 ± 1.3)	72.1 ± 17.4 (4.4 ± 1.2)	0.0015
(l)	(4.6 ± 1.2)			0.0504
DL_*CO*_% predicted value	44.9 ± 16	50.2 ± 15.2	38.6 ± 14.7	<0.0001
KCO% predicted value	72.8 ± 19.9	77 ± 17.7	67.6 ± 21.4	0.0013
**HRCT scan pattern**				
Typical UIP (%)	199 (75.6)	110 (79.1)	89 (71.7)	0.3917
Probable UIP (%)	64 (24.3)	29 (20.8)	35 (28.2)	0.3917
Emphysema (%)	70 (26.6)	36 (25.8)	34 (27.4)	0.7820

*Comparison between pirfenidone and nintedanib subgroups. ILD, interstitial lung disease; PFTs, pulmonary function tests; FVC, forced vital capacity; FEV1, forced expiratory volume in the first second; TLC, total lung capacity; DL_CO_, lung diffusion capacity for carbon monoxide; KCO, carbon monoxide transfer coefficient; HRCT, high resolution computed tomography; UIP, usual interstitial pneumonia.

Fifty-six patients (21.2%, 43 treated with nintedanib) showed advanced stage disease at diagnosis and were treated on compassionate grounds (pirfenidone FVC < 50% and/or DLco < 35%; nintedanib FVC < 50% and DLco < 35% of predicted values). Twenty-two (8.3%) of them had been on long-term oxygen therapy (LTOT) due to chronic respiratory failure before starting antifibrotic treatment.

The study population was stratified by antifibrotic treatment: 139 patients (107 males) treated with pirfenidone, 124 (96 males) treated with nintedanib. Patients treated with nintedanib were significantly older (*p* < 0.0001) than the pirfenidone group at the start of antifibrotic therapy and showed worse FVC and DLco percentages (*p* = 0.0004 and *p* < 0.0001, respectively).

### Outcome Analysis

**FIGURE 1:**
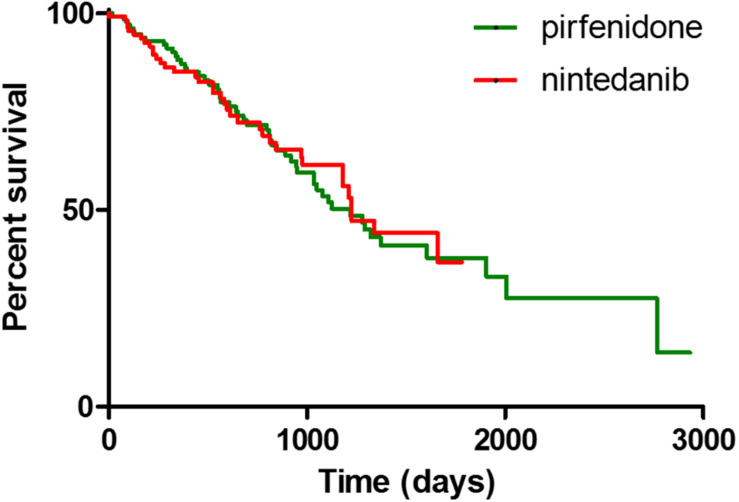
Kaplan Meier curves for survival analysis between IPF patients treated with pirfenidone and nintedanib.

At 1st January 2020 (885.3 ± 559.5 days of observation), median of survival was 1224 days. During follow-up, 59 patients died (22.4%, 29 pirfenidone, 30 nintedanib), 7 patients underwent lung transplant (2.6%, 6 pirfenidone, 1 nintedanib) and 21 (7.9%) discontinued treatment due to incoercible drug-related adverse effects (14 pirfenidone, 7 nintedanib). No significant differences in mortality rate were found between treatment groups (log rank test 0.02333, *p* = 0.8786) (Figure 1).

Concerning functional deterioration, mean time to FVC decline > 10% (TTD10) was 495.3 ± 263 days in the overall population. Pre-treatment LFTs and DLco were available in 66 and 52 patients, 44 on pirfenidone, 22 on nintedanib, respectively. In general, FVC and DLco reduction rates slowed significantly after 1 year of antifibrotic therapy (*p* = 0.0062 and *p* = 0.0075, respectively). Accordingly, the percentage of patients reaching TTD10 was significantly higher in the pre-treatment period than after 1 year of therapy (*p* = 0.0012). FVC and DLco reduction rates during follow-up are reported in Table 2. In the following years of therapy, FVC progression rate tended to accelerate, even though not reaching statistical significance (*p* = 0.0567).

**TABLE 2:** FVC and DLCO progression rate of IPF patients in pretreatment period, at baseline (initiation of antifibrotic therapy) and during the follow-up.

Parameters	Pre-treatment	Baseline (time 0)	12 months	24 months	36 months	48 months	60 months
Days from time 0	370.5 ± 100.4		363.7 ± 62.8	717.4 ± 98.5	1083.4 ± 89.6	1447.9 ± 107.5	1790.6 ± 58.2
Pirfenido ne (*n*°)	44	139	134	87	40	21	7
Nintedani b (*n*°)	22	124	101	54	27	13	5
FVC%	82.7 ± 21.4	78.2 ± 20.1	78.1 ± 22.3	77.6 ± 22.5	76.4 ± 22	77.8 ± 22.3	72.8 ± 12.7
ΔFVC%*	+6.4 ± 9.1		−2.4 ± 8.1	−5.9 ± 11.2	−8.4 ± 12.1	−11.7 ± 12.8	−16.6 ± 9.9
FVC (ml)	2667.3 ± 838	2540.8 ± 79	2463.3 ± 824	2395.3 ± 871.8	2173.6 ± 780.5	2089.5 ± 714.1	2416 ± 542.9
ΔFVC (ml)*	+202.5 ± 27.1	4.4	−90.6 ± 244	−209.8 ± 329.3	−269.2 ± 266.6	−296.8 ± 292.4	−534 ± 296.7
DL_*CO*_%	45.7 ± 18.3	44.9 ± 16	44.2 ± 17.4	44.3 ± 17.2	42.6 ± 16.6	36.2 ± 10.5	35.3 ± 8.5
ΔDL_*CO*_%*	+5.4 ± 7.7		−3.8 ± 9.3	−8.2 ± 10.8	−11.6 ± 11.8	−18.2 ± 8.1	−17.2 ± 7.6

*Difference against baseline values. FVC, forced vital capacity; FEV1, forced expiratory volume in the first second; DL_CO_, lung diffusion capacity for carbon monoxide.

**FIGURE 2:**
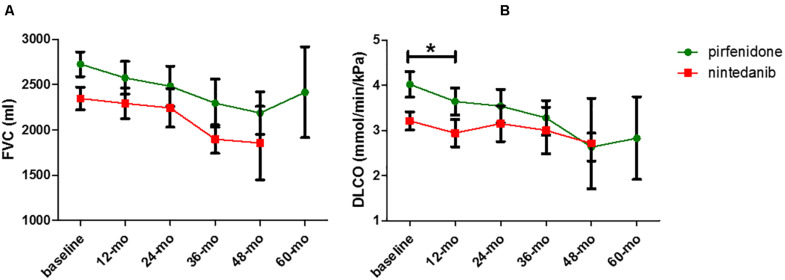
Comparison of FVC **(A)** and DL_*CO*_ **(B)** decrease rate between pirfenidone and nintedanib subgroup (*p = 0.0201).

**FIGURE 3:**
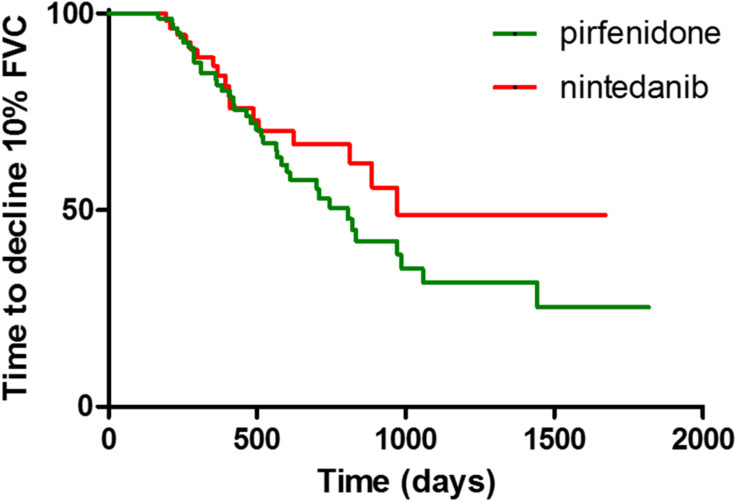
Difference of time to decline of FVC > 10% between pirfenidone and nintedanib subgroups, expressed with Kaplan-Meier curves.

Concerning comparisons between the pirfenidone and nintedanib groups, no statistically significant difference in terms of FVC deterioration rate (*p* = 0.9396 and *p* = 0.6907 for absolute and percentage values, respectively) (Figure 2) and TTD10 was observed during follow-up (log rank test 1.903, *p* = 0.1677) (Figure 3). After 1 year of antifibrotic treatment, we observed a more pronounced decrease in DLco in patients treated with pirfenidone than in those treated with nintedanib (*p* = 0.0167 and *p* = 0.0201 for absolute and percentage values), but this significant difference no longer emerged in subsequent years of follow-up (Figure 2).

Concerning tolerability, pirfenidone and nintedanib were both generally well tolerated: most adverse effects were mild to moderate and were readily managed with temporary discontinuation of therapy or with supporting treatments (Table 3). No drug-related fatal events were reported.

**TABLE 3:** Adverse events (AE) reported in our cohort.

Side effects	Pirfenidone	Nintedanib
***N*° of patients with AE (%)**	44 (31)	52 (41.9)
**Interruption of treatment (%)**	14 (10)	7 (5.6)
**Hypertransaminasemia (%)**	7 (5)	12 (9.6)
Time from start treatment (months)	4.2 ± 7.4	3.5 ± 6.3
NCI-CTC grade 1	6	5
NCI-CTC grade 2	1	5
NCI-CTC grade 3	0	1
NCI-CTC grade 4	0	1
NCI-CTC grade 5	0	0
**Anorexia (%)**	10 (7.2)	3 (2.4)
Time from start treatment (months)	6.4 ± 5.2	4.9 ± 4.1
NCI-CTC grade 1	7	3
NCI-CTC grade 2	3	0
NCI-CTC grade 3	0	0
NCI-CTC grade 4	0	0
NCI-CTC grade 5	0	0
**Weight loss (%)**	25 (17.9)	17 (13.7)
Time from start treatment (months)	4.1 ± 6.6	4.8 ± 5.1
NCI-CTC grade 1	18	15
NCI-CTC grade 2	7	2
NCI-CTC grade 3	0	0
NCI-CTC grade 4	0	0
NCI-CTC grade 5	0	0
**Nausea (%)**	10 (7.2)	5 (4)
Time from start treatment (months)	2.9 ± 4.4	2.5 ± 2
NCI-CTC grade 1	8	2
NCI-CTC grade 2	2	3
NCI-CTC grade 3	0	0
‘NCI-CTC grade 4	0	0
NCI-CTC grade 5	0	0
**Pyrosis (%)**	13 (9.3)	9 (7.2)
Time from start treatment (months)	2.8 ± 3.9	2.5 ± 2.8
NCI-CTC grade 1	10	5
NCI-CTC grade 2	3	4
NCI-CTC grade 3	0	0
NCI-CTC grade 4	0	0
NCI-CTC grade 5	0	0
**Diarrhea (%)**	3 (2.1)	44 (31.6)
Time from start treatment (months)	5.2 ± 4.8	2.2 ± 3.1
NCI-CTC grade 1	1	20
NCI-CTC grade 2	2	18
NCI-CTC grade 3	0	6
NCI-CTC grade 4	0	0
NCI-CTC grade 5	0	0
**Constipation (%)**	7 (5)	2 (1.6)
Time from start treatment (months)	4.6 ± 6.7	5.1 ± 3.2
NCI-CTC grade 1	5	2
NCI-CTC grade 2	2	0
NCI-CTC grade 3	0	0
NCI-CTC grade 4	0	0
NCI-CTC grade 5	0	0
**Photosensitivity (%)**	16 (11.5)	0
Time from start treatment (months)	3.2 ± 5.1	
NCI-CTC grade 1	0	
NCI-CTC grade 2	4	
NCI-CTC grade 3	12	
NCI-CTC grade 4	0	
NCI-CTC grade 5	0	
**Skin rash (%)**	19 (13.6)	2 (1.6)
Time from start treatment (months)	2.6 ± 4.5	3.6 ± 4.2
NCI-CTC grade 1	9	0
NCI-CTC grade 2	10	2
NCI-CTC grade 3	1	0
NCI-CTC grade 4	0	0
NCI-CTC grade 5	0	0
**Itch (%)**	2 (1.4)	2 (1.6)
Time from start treatment (months)	6.7 ± 4.3	8.1 ± 5.4
NCI-CTC grade 1	0	1
NCI-CTC grade 2	1	1
NCI-CTC grade 3	1	0
NCI-CTC grade 4	0	0
NCI-CTC grade 5	0	0
**Dizziness (%)**	1 (0.7)	0
Time from start treatment (months)	1.5	
NCI-CTC grade 1	0	
NCI-CTC grade 2	1	
NCI-CTC grade 3	0	
NCI-CTC grade 4	0	
NCI-CTC grade 5	0	

*NCI-CTC, National Cancer Institute Common Terminology Criteria.*

## Discussion

This observational real-life study of patients with IPF treated with nintedanib and pirfenidone offers a comparative evaluation of drug effectiveness after a long follow-up. Clinical, functional and survival data was collected for 60 months at our Regional Referral Center for Rare Lung Diseases, ensuring the homogeneity of data collection and the clinical management of patients. Although monocentric, this is the first real-life study to evaluate the safety and efficacy of the two antifibrotic treatments for a significantly longer follow-up than those reported in the international clinical trials and observational cohorts (Noble et al., 2011; King et al., 2014; Richeldi et al., 2014; Margaritopoulos et al., 2018). Only four previous studies have investigated the efficacy of pirfenidone over an observation period longer than 2 years in a real-life setting (Bando et al., 2016; Tzouvelekis et al., 2017; Zurkova et al., 2019; Vietri et al., 2020). The only evidence available for nintedanib comes from open-label extension studies of clinical trials; to our knowledge, no real-life data is available beyond 3 years of treatment (Richeldi et al., 2018; Crestani et al., 2019; Antoniou et al., 2020; Song et al., 2020).

As expected, our population showed a clear predominance of males and most patients were former smokers. The prevalence of males and smoking in our population, as well as the mean age of patients (over 65 years), are in line with epidemiological studies (Fernández-Fabrellas et al., 2019; Tran et al., 2020). Due to Italian prescription criteria for antifibrotic drugs, the patients treated with nintedanib were older than those treated with pirfenidone and had worse FVC and DLco percentage at baseline. This discrepancy is also related to the inclusion of patients treated with nintedanib on compassionate grounds, which allowed us to recruit and treat patients in an advanced stage of disease. Moreover, 8% of our patients were on oxygen therapy before starting antifibrotic treatment, in substantial conformity with other real-file IPF studies (Sköld et al., 2016; Bargagli et al., 2019). It is surely interesting that, despite the age discrepancy, no significant differences were found between pirfenidone and nintedanib subgroups in terms of progression-free survival and mortality. These findings further support the substantial efficacy of antifibrotic drugs and specifically of nintedanib, also in elderly age, as already reported in literature (Takeda et al., 2020).

Our results confirm the effectiveness of pirfenidone and nintedanib in slowing disease progression in terms of FVC reduction rate and in maintaining the effect in subsequent years of treatment. This result was emphasized by comparison of functional deterioration in the years before and after antifibrotic therapy, which showed a substantial halving of the FVC and DL_*CO*_ reduction rates. Similar results were recently reported by Vietri et al. (2020) for pirfenidone in a cohort of IPF patients. In our study, both antifibrotic molecules stabilized functional deterioration over many years of follow-up, suggesting their long-term effectiveness. Moreover, the deterioration of FVC and DL_*CO*_ was stabilized in the older population with advanced disease, further confirming previous studies that demonstrated that pirfenidone and nintedanib are also effective in advances stages of IPF (Wuyts et al., 2016; Nathan et al., 2019). Interestingly, the decrease in DLco was more pronounced after 1 year of pirfenidone than after 1 year of nintedanib treatment, although the significance tended to disappear after the first year of follow-up. Despite the limitations of our retrospective study, our results may suggest that nintedanib has a specific effect on slowing the decline in lung diffusion capacity with respect to pirfenidone, probably due to its well-known antiangiogenic properties (Hilberg et al., 2008; Reck, 2015). However, our nintedanib group showed older age and worse FVC and DLco values, aspects that likely influence our analysis, together with the relatively small sample size. Nevertheless, our results are certainly interesting and merit full assessment in a prospective study with larger cohorts.

Concerning mortality, our data showed a median survival of 1224 days, similar to that of previous reports (Margaritopoulos et al., 2018; Antoniou et al., 2020; Vietri et al., 2020). Despite the absence of a control group, comparison of our data with survival data of historical cohorts of patients reported in the literature confirmed the efficacy of nintedanib and pirfenidone in prolonging life expectancy in patients with IPF (Costabel et al., 2017; Fisher et al., 2017; Margaritopoulos et al., 2018; Lancaster et al., 2019). No significant differences were found in terms of survival between the pirfenidone and nintedanib groups. Although our data may be influenced by demographic discrepancies between the two groups, our results suggest that the two drugs have substantially the same performance in preventing functional deterioration and reducing mortality. These assumptions are in line with a recent paper by Cerri et al. (2019), although our study considered a larger cohort of patients (including those with advanced disease) and a much longer follow-up, further underlining the reliability of both antifibrotic drugs in the treatment of IPF.

Regarding drug tolerability, our results are in line with previous reports (Lancaster et al., 2017; Rodríguez-Portal, 2018). Despite the high prevalence of side effects (especially weight loss with pirfenidone and diarrhea with nintedanib), most (>90%) were mild and easily managed with supporting therapies and temporary dose reductions or discontinuation. No fatal events were reported in our cohort, confirming the good safety profile of the two drugs even in fragile patients, over a 5 year observation period.

In conclusion, this real-life retrospective comparison of the long-term effectiveness of the two currently approved pharmacological treatments for IPF showed similar efficacy in reducing functional deterioration and improving life expectancy, associated with acceptable tolerability, even in subjects with greater functional impairment. Long-term multicenter prospective studies, including populations of patients excluded from randomized clinical trials, are needed to confirm our findings. Future research will necessarily focus on identifying biomarkers to predict response to a specific drug, to enable a personalized therapeutic approach for IPF patients.

## Data Availability Statement

The raw data supporting the conclusions of this article will be made available by the authors, without undue reservation, to any qualified researcher.

## Ethics Statement

The studies involving human participants were reviewed and approved by the Comitato Etico Area Vasta Sud Est (C.E.A.V.S.E.). The patients/participants provided their written informed consent to participate in this study.

## Author Contributions

PC, RR, and EB: conception and study design, interpretation of results, writing of the manuscript. LB, Md’A, VA, and CM: data acquisition and analysis, revision of the study, interpretation of results. PC, EB, PR, and PS: statistical analysis, revision of the study. All authors approved the final version of the study and agreed to be accountable for all aspects of the work in ensuring that questions related to the accuracy or integrity of any part of the work are appropriately investigated and resolved.

## Conflict of Interest

The authors declare that the research was conducted in the absence of any commercial or financial relationships that could be construed as a potential conflict of interest.

## Abbreviations

DL_*co*_: diffusing capacity of the lung for CO
FEV1: forced expiratory volume in 1 second
I FVC: forced vital capacity
HRCT: high resolution computed tomography
PF: idiopathic pulmonary fibrosis.
